# BsmI, ApaI and TaqI Polymorphisms in the Vitamin D Receptor Gene (*VDR)* and Association with Lumbar Spine Pathologies: An Italian Case-Control Study

**DOI:** 10.1371/journal.pone.0155004

**Published:** 2016-05-05

**Authors:** Alessandra Colombini, Marco Brayda-Bruno, Giovanni Lombardi, Samantha Jennifer Croiset, Cristina Ceriani, Cinzia Buligan, Mattia Barbina, Giuseppe Banfi, Sabina Cauci

**Affiliations:** 1 Laboratory of Experimental Biochemistry and Molecular Biology, I.R.C.C.S. Istituto Ortopedico Galeazzi, Milan, Italy; 2 Scoliosis Unit, Department of Orthopedics and Traumatology – Spine surgery III, I.R.C.C.S. Istituto Ortopedico Galeazzi, Milan, Italy; 3 Department of Medical and Biological Sciences, University of Udine, Udine, Italy; 4 Vita-Salute San Raffaele University, Milan, Italy; University of Birmingham, UNITED KINGDOM

## Abstract

Three adjacent single nucleotide polymorphisms of the vitamin D receptor gene (*VDR*) BsmI (rs1544410), ApaI (rs7975232), and TaqI (rs731236) are commonly studied in several pathologies. We aimed to evaluate the distribution of *VDR* BsmI, ApaI, and TaqI allele, genotype, and haplotype frequencies in an Italian cohort of 266 patients with lumbar spine disorders assessed by Magnetic Resonance Imaging and 252 asymptomatic controls. The exposure to putative risk factors was evaluated by a questionnaire. Polymorphisms were detected by PCR-RFLP and TaqMan^®^ SNP Genotyping Assay. The results were statistically adjusted for the identified conventional risk factors. The three SNPs were in linkage disequilibrium. For all cases *BbAaTT* was a 3-fold risk factor OR = 3.38), whereas *bbAATT* (OR = 0.22), and *bbaaTT* (OR = 0.47) genotypes were found to be protective. Specifically, for patients affected by disc herniation only (n = 88) and all lumbar pathologies excluding stenosis and/or spondylolistesis (n = 215) *B* allele, *Bb*, *Aa*, and *BbAaTT* genotypes were risky, whereas *b* allele, *bb*, *aa*, and *bbaaTT* genotypes were protective. In patients affected by osteochondrosis with or without disc hernation (n = 50), *T* allele, *Aa*, and *bbAaTT* genotypes were risky, whereas *t* allele, *AA*, *tt* genotypes were protective. In patients affected by stenosis and/or spondylolistesis (n = 51) no significant associations were found. This is the first study showing an association of the three genetic *VDR* variants BsmI, ApaI, and TaqI and lumbar spine pathologies. Our study contributes to delineate genetic risk factors for specific subgroups of patients with lumbar spine pathologies highlighting the importance of haplotype analysis, and of detailed clinical evaluation of the patients for identification of genetic biomarkers.

## Introduction

BsmI (rs1544410), ApaI (rs7975232), and TaqI (rs731236) are adjacent restriction fragment length polymorphisms (RFLP) located in the region of intron 8/exon 9 of the vitamin D receptor gene (*VDR*), which has been commonly associated with several diseases [1]. Furthermore, they have been also associated with lumbar disc degeneration (LDD) [2–4]. These three single nucleotide polymorphisms (SNPs), BsmI (G>A), ApaI (T>G), and TaqI (T>C), are located close to the 3’ terminus of the gene, and do not determine structural modification in the receptor [1]. There is no clear evidence about the effect of BsmI and ApaI variants in affecting the VDR activity, whereas TaqI is a synonymous SNP [1]. Nevertheless, some authors hypothesized some effects on mRNA stability [1,5]. These 3 SNPs were frequently reported to be in linkage disequilibrium (LD). Moreover, it has been suggested that they could be in LD with other polymorphisms affecting the VDR function, this could probably explains their association with several pathological phenotypes [1].

The majority of previous studies dealing with LDD focused specifically on the TaqI polymorphism. The first association between TaqI SNP and intervertebral disc disease was reported in Finnish males [6]. Men with the *tt* genotype had the lowest prevalence of disc bulges and fewer lumbar disc herniation in comparison with those with the *TT* genotype [7]. On the contrary, other three Finnish studies found no significant differences in the genotype frequencies of *VDR* TaqI between pathological and asymptomatic men, no significant association of the SNP with Modic changes [8,9] and with lumbar spinal stenosis [10].

Other studies in white Caucasian subjects analyzing TaqI SNP were conducted in Turks [11,12], Norwegians [13], and Australians [14]. In young Turks no significant differences were observed in TaqI allele frequencies between patients with hernia or disc degeneration and controls. However, the *TT* genotype was associated with milder forms, while *tt* genotype with more severe forms of disc degeneration. Specifically, *TT* and *Tt* genotypes were associated with disc herniation protrusion, whereas the *tt* genotype was associated with extrusion/sequestration [11]. Another study in Turks showed that the presence of *VDR* TaqI mutation was associated with a worse disc degeneration score, and with the development of lumbar disc degeneration [12].

A decrease in the presence of spinal osteophytosis and disc space narrowing from *tt* to *TT* genotypes was observed in Australians [14]. On the contrary, in a Norway case-control study no association between TaqI SNP and LDD was reported [13].

Three studies on the association of TaqI SNP with LDD were found in Asians [15–17]. In young Japanese the *Tt* genotype was associated with an increased risk of severe, multilevel disc degeneration and herniation and with an early age of the disease onset [15]. In contrast, the frequencies of *T* and *t* alleles was not associated with the presence of disc degeneration and osteophyte in elderly Japanese females [16]. In a Chinese study, involving individuals younger than 40 years, the *t* allele was associated with degenerative disc features and disc bulging [17].

Taking together these results concerning TaqI SNP showed no association of this SNP with LDD in more than half studies (6/10). Moreover inconsistent findings between studies were reported, showing an association of *TT* and *tt* genotypes in Caucasians, *Tt* genotype and *t* allele in Asians with signs of disc degeneration.

To our knowledge, the great majority of studies concerning lumbar spine pathologies analyzed the association with TaqI and FokI *VDR* polymorphisms [18], only 4 studies analyzed ApaI [15,19–21] and only 2 studies BsmI polymorphism [20,22]. None of the above mentioned studies performed the analysis of all the 3 RFLP BsmI, ApaI and TaqI polymorphisms at the same time.

The *A* allele of *VDR* ApaI was significantly associated with LDD with synergistic interaction between the allele and spine bending/twisting in a Chinese case-control study involving low back pain patients [19]. Recently, Zawilla et al. found an OR = 3.1 for the association of mutant ApaI and LDD [21]. Moreover, *B* allele of *VDR* BsmI and birthweight were associated with increasing severity of osteophyte grade in British men with lumbar spine osteoarthritis [22].

Concerning haplotypes, the BsmI-ApaI haplotypes were evaluated in Japanese postmenopausal females affected by lumbar spondylosis [20]. This study observed an association of BsmI-ApaI *VDR* haplotypes with the severity of the pathology that was more significant in the older (>63.6 years) than in the younger subjects [20]. However, differently from the majority of the studies, in the Japanese study the BsmI and ApaI RFLPs were not in LD.

Differences in ethnicity, inclusion criteria when comparing the same ethnic group, and a not clear definition of the clinical phenotypes are likely responsible for the inconsistent results obtained so far. It is also to note that the extent of LD among BsmI, ApaI and TaqI varies in different studies [1], so that the choice to examine only one polymorphism (e.g., TaqI) as representative of the other two polymorphisms can possibly be misleading.

Detailed analysis of specific subgroups of LDD patients was performed in an Italian case-control study published in 2014 by our group [18]. In that study the *FF* genotype and *F* allele of the *VDR* FokI SNP were associated with an approximately 2-fold risk to develop discopathies, and particularly discopathies and/or osteochondrosis concomitant with disc herniation [18]. Further, major gender-related effects were observed [23,24].

By analyzing the same cohort of LDD subjects of our previous studies [18, 23], the aim of the present investigation was to evaluate the distribution of *VDR* BsmI, ApaI and TaqI allele, genotype, and haplotype frequencies in patients with specifically defined lumbar spine pathologies in comparison with asymptomatic controls. To our knowledge this is the first study evaluating at the same time all the 3 adjacent polymorphisms in relation to LDD.

## Material and Methods

### Ethics Statement

The study was approved by the Institutional Review Board ASL Città di Milano, protocol number GENODISC01. The methods used in this study were in accordance with the Helsinki Declaration of 1975 as revised in 1996.

### Subjects and clinical assessment

Based on a case-control design, 266 patients (outpatients or hospitalized) with lumbar spine disorders recruited for the European Genodisc Project, and 252 asymptomatic controls (most were healthy volunteers, some were blood donors, few were subjects hospitalized for anterior cruciate ligament injuries or hallux valgus surgery) were enrolled at the I.R.C.C.S. Istituto Ortopedico Galeazzi (Milan, Italy) as previously described [18]. In respect to our previous investigation [18], the number of controls was increased including 32 additional subjects to have a total number of controls close to cases.

All subjects signed a written informed consent. Cases and controls were enrolled from May 2009 to December 2013. Exclusion criteria for both cases and controls were: pathologic condition such as cervical discopathies, scoliosis, fibromyalgia, pregnancy at study enrollment, and chronic diseases like diabetes, cardiovascular diseases, malignancies, lupus erythematosus, and rheumatoid arthritis. Assessment of lumbar spine disorders and patient’s classification into 4 different mutually exclusive subgroups (designed from 1 to 4 subgroup) based on detailed diagnosis were performed by a clinician expert in spine diseases by contrast-enhanced MRI 12 scans of the lumbar spine with a 1.5 T scanner (Avanto, Siemens, Erlangen, Germany) as described in our previous paper [18].

Subgroup 1 comprised patients affected by disc herniation only; Subgroup 2 comprised patients affected by osteochondrosis associated or not with disc hernation; Subgroup 3 comprised patients affected by discopathies, osteochondrosis or both without disc herniation; Subgroup 4 comprised patients affected by stenosis, lytic/isthmic spondylolisthesis, and degenerative spondylolisthesis.

Due to the close linkage between discopathies, disc herniation and osteochondrosis, a further subgroups division from A to D (not mutually exclusive) was performed: Subgroup A, comprising all herniation cases i. e. Subgroup 1 grouped with Subgroup 2; Subgroup B, including all discopathies and/or osteochondrosis regardless of herniation, i.e. Subgroup 2 grouped with Subgroup 3; Subgroup C, comprising all discopathies concomitant with disc herniation grouped with subjects with discopathies alone; and Subgroup D, comprising all osteochondrosis concomitant with disc herniation grouped with subjects with osteochondrosis alone.

Table 1 reported information for all study subjects including family medical history (parents, brothers or sisters) about spine disorders, smoking habit, job physical demand for the majority of the working years (evaluated by the following score: 0 = sedentary; 1 = light; 2 = medium; 3 = heavy), and hours/day spent driving or as a passenger in motorized vehicles (exposure to vibrations).

**Table 1:** Characteristics of the study subjects and comparison of conventional and non-genetic risk factors of lumbar spine pathologies between controls and all cases or subgroups.

Factors		Controls	All Cases	Subgroup 1	Subgroup 2	Subgroup 3	Subgroup 4
		n = 252	n = 266 (100%)	n = 88 (33.1%)	n = 87 (32.7%)	n = 40 (15.0%)	n = 51 (19.2%)
Age (Years)	mean ± SD	39.42±10.61	44.18±9.12***	42.36±9.30*	43.69±8.90***	42.80±8.33*	49.22±8.16***
Gender	Males n (%)	127 (50.4)	148 (55.6)	47 (53.4)	55 (63.2)*	21 (52.5)	25 (49.0)
	Females n (%)	125 (40.6)	118 (44.4)	41 (46.6)	32 (36.8)	19 (47.5)	26 (51.0)
BMI (kg/m^2^)	mean ± SD	24.19±3.78	25.29±4.06***	24.91±4.07	25.27±3.59**	24.29±3.91	26.75±4.61***
Family history	n (%)	37 (14.7)	97 (36.5)***	33 (37.5)***	38 (43.7)***	11 (27.5)*	15(29.4)*
Past and present smoker	n (%)	104 (41.3)	144 (54.1)**	53 (60.2)**	50 (57.5)**	16 (40.0)	25 (49.0)
Physical job demand^1^ (Score 0–3)	mean ± SD	1.06±1.00	1.37±1.08***	1.39±1.07*	1.38±1.11*	1.40±1.13	1.33±1.05
Exposure to vibrations (Hours/day)	mean±SD	1.40±1.16	2.20±2.56**	2.33±2.90	2.29±2.43**	2.25±2.80	1.78±1.90

^1^ 5 patients had missing information about intensity of physical demand at work, thus a total of 261 data were available. Physical job demand score used: 0 = sedentary; 1 = light; 2 = medium; 3 = heavy.

Subgroup 1 = patients with disc herniation alone; Subgroup 2 = patients with discopathies and/or osteochondrosis associated with disc herniation; Subgroup 3 = patients with discopathies and/or osteochondrosis without herniation; Subgroup 4 = patients with stenosis and/or spondylolisthesis.

* P<0.05,

** P<0.01,

*** P<0.001

### Determination of genotypes

Blood samples were collected from the antecubital vein with evacuated ethylenediamine tetra acetic acid (EDTA) tubes (Vacutainer Tubes, Becton-Dickinson, Franklin Lakes, NJ, USA) from cases and controls. Genomic DNA was extracted from white blood cells according to the procedure of the DNeasy Midi kit (Qiagen, Duesseldorf, Germany) as described [18]. Polymerase chain reaction and restriction fragment length polymorphism (PCR-RFLP) methods were applied to detect the BsmI, ApaI, and TaqI polymorphisms of *VDR*. Genomic DNA was amplified using PCR. At first DNA was denatured at 95°C for 5 minutes. Standard PCR conditions for DNA amplification using appropriate primers [25] were as follows: 94°C for 1 minute, annealing temperature 64°C for 1 minute and 72°C for 2 minutes for 32 cycles and finally 96°C for 1 minute and 72°C for 5 minutes.

**Fig 1:**
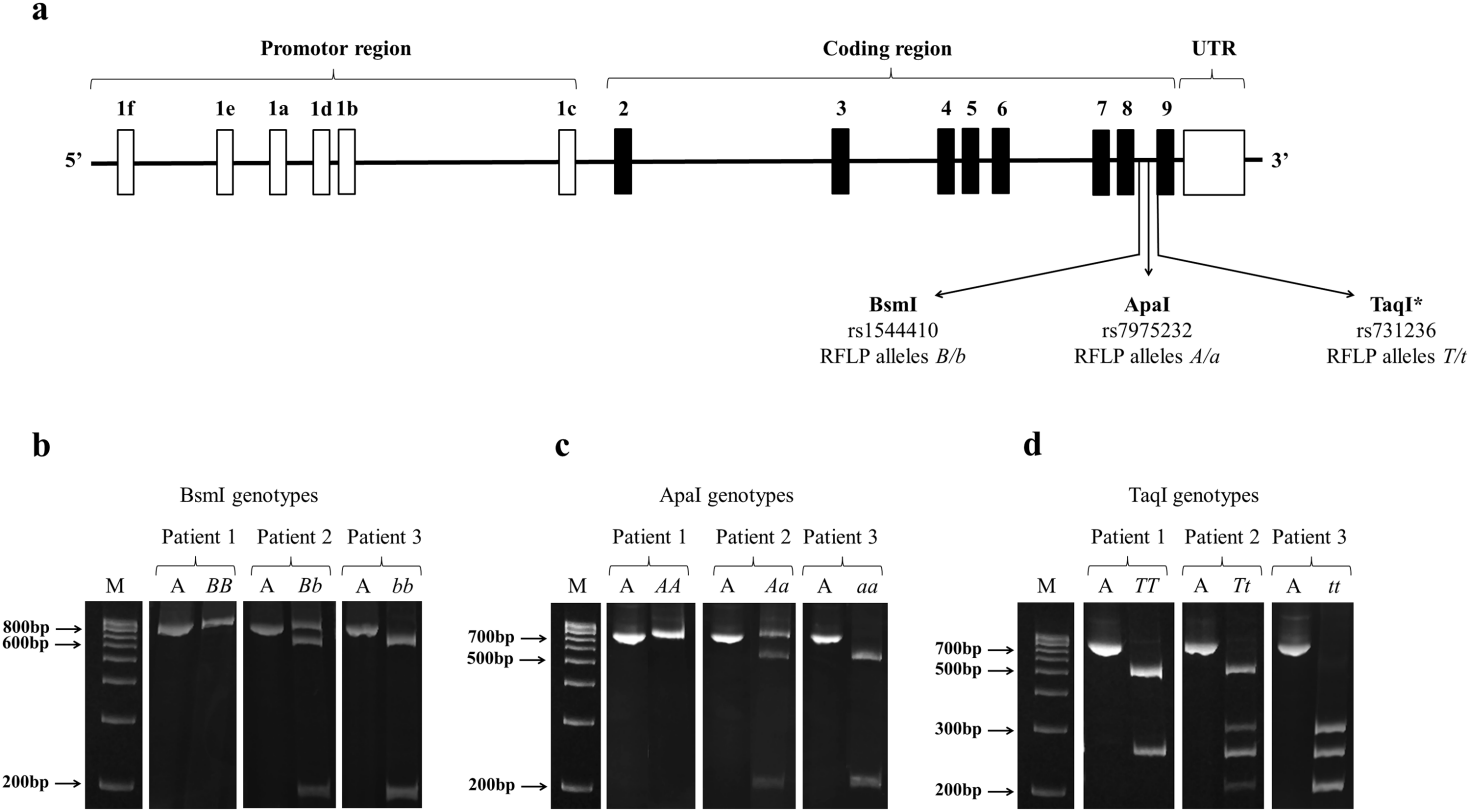
Structure of the genomic region of the *VDR* and location of BsmI, ApaI and TaqI (*in the coding sequence, exon 9) SNPs located near the 3’ terminus of the gene (a). Representative gels for the determination of BsmI (b), ApaI (c) and TaqI (d) genotypes in three patients are showed. In the first lane there is a molecular weight DNA ladder (M) for size estimation of the DNA fragments. The letter “A” indicates the PCR amplicons which are of 825 bp for BsmI and 740 bp for ApaI and TaqI. After digestion of the PCR product with BsmI restriction enzyme an undigested 825 bp fragment (homozygous genotype *BB*), partially digested 825, 650 and 175 bp fragments (heterozygous genotype *Bb*), or totally digested 650 and 175 bp fragments (homozygous genotype *bb*) are present (b). After digestion of the PCR product with ApaI restriction enzyme an undigested 740 bp fragment (homozygous genotype *AA*), partially digested 740, 530 and 210 bp fragments (heterozygous genotype *Aa*), or totally digested 530 and 210 bp fragments (homozygous genotype *aa*) are present (c). After digestion of the PCR product with TaqI restriction enzyme, digested 495 and 245 bp fragments (homozygous genotype *TT*), partially digested 495, 290, 245 and 205 bp fragments (heterozygous genotype *Tt*), or totally digested 290, 245 and 205 bp fragments (homozygous genotype *tt*) are present (d). All the images of the original gels from which we cropped the representative images showed in Fig 1 are available as S1–S6 Figs.

The BsmI, ApaI and TaqI polymorphisms of *VDR* were studied using previously tested primers [25] to amplify the first part of intron 8 for BsmI, and a fragment of intron 8/exon 9 for ApaI and TaqI (Fig 1). The allele digested by the restriction enzyme was denoted by a lower letter, whereas that not digested was indicated by a capital letter.

To analyse the BsmI polymorphism, the resulting amplified 825 bp PCR fragment was digested with BsmI restriction enzyme (Euroclone, Milano, Italy) generating two fragments of 650 bp and 175 bp in presence of the *b* allele. Since a mismatched base at the primer binding region can lead to a drop out of *b* allele during PCR amplification and to the false higher prevalence of *BB* genotype [26], data obtained with PCR-RFLP were confirmed using TaqMan^®^ SNP Genotyping Assay (Life Technologies, Carlsbad, CA, USA) for rs1544410. A higher number of *Bb* subjects with a proportionally smaller number of *BB* subjects were observed after application of the genotyping assay. Specifically, out of the 141 subjects having the *BB* genotype assigned with PCR-RFLP, 48 subjects were assigned as *Bb*, and 2 as *bb* after the analysis with the TaqMan^®^ SNP Genotyping Assay.

ApaI digestion of the 740 bp amplified DNA used to determine both ApaI and TaqI RFLP, resulted in two fragments of 530 bp and 210 bp in presence of the *a* allele.

Digestion with TaqI of the 740 bp PCR fragment resulted in three fragments of 290 bp, 245 bp and 205 bp in presence of the *t* allele, and in two fragments of 495 bp and 245 bp in its absence (*T* allele) due to an additional monomorphic TaqI site.

DNA fragments were separated by electrophoresis on polyacrylamide gel.

Genotypes were designated by a lowercase letter (*b*, *a*, *t* alleles) for the presence of the restriction enzyme site, and by a capital letter (*B*, *A*, *T* alleles) for its absence.

### Statistical analysis

Kolmogorov-Smirnov and Shapiro-Wilk tests were used to assess the normality of data distribution. Since data were not normally distributed, Mann Whitney test were used to assess the differences between continuous variables such as age, BMI, physical job demand, and exposure to vibration in all cases or subgroups and controls. For categorical variables, Odds ratios (ORs) and the 95% confidence interval (CI) were calculated to evaluate the risk of spine pathologies by comparing cases or subgroups of patients with controls, significance was evaluated by chi square Pearson or Fisher exact test, as appropriate (for clarity we showed in Table 1 only significance and not all ORs and CIs).

Tests for deviations from Hardy-Weinberg equilibrium (HWE) were performed using a chi-square distribution in all the subjects, and in cases and controls separately for each SNP.

ORs and the CI were calculated to set the association between alleles, genotypes, and haplotypes and risk of spine pathologies in cases, controls and subgroups of patients. Logistic regression was used to evaluate effects of confounders by obtaining adjusted ORs and 95% CIs for genotypes and alleles. Adjusted analysis included conventional risk factors previously observed [18] and confirmed in this cohort such as: age, BMI, smoke, physical job demand and exposure to vibrations (Table 1). Family history was not included as confounder to avoid overcorrection by considering that it includes genetic hereditary components. Throughout the text adjusted ORs for the genetic associations were reported.

Eight rare BsmI-ApaI-TaqI haplotypes estimated to be less than 3% in frequency in both cases and controls (including in total 11 subjects) were not reported. LD between the SNPs was determined using Haploview [27]. Haplotype frequencies were estimated with the program CHAPLIN [28].

Significance level was held at 0.05, and P values ≤ 0.10 were considered as a tendency. Statistical softwares used were GraphPad Prism version 5.00 (GraphPad software, La Jolla, CA, USA), and SPSS version 14.0 (SPSS Inc., Chicago, IL, USA).

## Results

### *VDR* BsmI, ApaI, TaqI genotypes and alleles

In our total sample the frequency of *BB* was 17.6% (91/518), *Bb* was 51.3% (266/518), and *bb* was 31.1% (161/518). *B* allele had a frequency of 43.2% (448/1036), and the *b* allele had a frequency of 56.8% (588/1036). The observed BsmI genotype frequencies were in HWE (X^2^ = 1.102, P = 0.29). In particular, controls did not deviate (X^2^ = 0.943, P = 0.33), while cases (X^2^ = 6.043, P = 0.01) deviated from HWE. The frequency of *AA* genotype was 34.0% (176/518), *Aa* was 48.1% (249/518), and *aa* was 17.9% (93/518). *A* allele had a frequency of 58.0% (601/1036), and the *a* allele had a frequency of 42.0% (435/1036). The observed ApaI genotype frequencies were consistent with HWE (X^2^ = 0.091, P = 0.76). Neither controls (X^2^ = 3.67, P = 0.05) nor cases (X^2^ = 2.09, P = 0.15) deviated from HWE. The frequency of *TT* genotype was 42.5% (220/518), *Tt* was 43.6% (226/518), and *tt* was 13.9% (72/518). *T* allele had a frequency of 64.3% (666/1036), and the *t* allele had a frequency of 35.7% (370/1036). The observed TaqI genotype frequencies were consistent with HWE (X^2^ = 1.287, P = 0.26). Neither controls (X^2^ = 1.058, P = 0.30) nor cases (X^2^ = 0.33, P = 0.56) deviated from HWE.

In S1 Appendix were reported the frequencies of BsmI, ApaI and TaqI genotypes in cases and controls.

Tables 2 and 3, respectively, illustrated frequencies of BsmI, ApaI and TaqI genotypes and alleles in controls compared with overall LDD cases, and specific subgroups of patients.

**Table 2:** Association of lumbar spine pathologies and *VDR* BsmI, ApaI, and TaqI genotypes.

	*BB*	*Bb*	*bb*	*AA*	*Aa*	*aa*	*TT*	*Tt*	*tt*
Controls n = 252 (%)	45 (17.9)	114 (45.2)	93 (36.9)	92 (36.5)	108 (42.9)	52 (20.6)	106 (42.1)	109 (43.2)	37 (14.7)
Cases n = 266 (%)	46 (17.3)	152 (57.1)**	68 (25.6)**	84 (31.6)	141 (53.0)*	41 (15.4)^	114 (42.9)	117 (44.0)	35 (13.1)
Subgroup 1 n = 88 (%)	15 (17.0)	54 (61.4)**	19 (21.6)**	26 (29.5)	51 (58.0)*	11 (12.5)*	37 (42.0)	40 (45.5)	11 (12.5)
Subgroup 2 n = 87 (%)	12 (13.8)	46 (52.9)	29 (33.3)	23 (26.4)^	50 (57.5)^	14 (16.1)	43 (49.4)	34 (39.1)	10 (11.5)
Subgroup 3 n = 40 (%)	9 (22.5)	23 (57.5)^	8 (20.0)*	16 (40.0)	20 (50.0)	4 (10.0)	12 (30.0)	21 (52.5)	7 (17.5)
Subgroup 4 n = 51 (%)	10 (19.6)	29 (56.9)	12 (23.5)^	19 (37.3)	20 (39.2)	12 (23.5)	22 (43.1)	22 (43.1)	7 (13.8)
Subgroup 1+2+3 n = 215 (%)	36 (16.7)	123 (57.2)**	56 (26.0)*	65 (30.2)	121 (56.3)**	29 (13.5)*	92 (42.8)	95 (44.2)	28 (13.0)
Subgroup A n = 175 (%)	27 (15.4)	100 (57.2)*	48 (27.4)*	49 (28.0)^	101 (57.7)**	25 (14.3)*	80 (45.7)	74 (42.3)	21 (12.0)
Subgroup B n = 127 (%)	21 (16.5)	69 (54.3)^	37 (29.1)^	39 (30.7)	70 (55.1)^	18 (14.2)	55 (43.3)	55 (43.3)	17 (13.4)
Subgroup C n = 64 (%)	17 (26.6)	34 (53.1)	13 (20.3)*	27 (42.2)	28 (43.7)	9 (14.1)	22 (34.4)	27 (42.2)	15 (23.4)^
Subgroup D n = 50 (%)	3 (6.0)^	26 (52.0)	21 (42.0)	7 (14.0)**	34 (68.0)**	9 (18.0)	29 (58.0)^	21 (42.0)	0 (0)*

Subgroup 1 = patients with disc herniation alone; Subgroup 2 = patients with discopathies and/or osteochondrosis associated with disc herniation; Subgroup 3 = patients with discopathies and/or osteochondrosis without herniation; Subgroup 4 = patients with stenosis and/or spondylolisthesis.

Subgroup A, Subgroup 1 grouped with Subgroup 2 (i.e. all hernia cases with or without concomitant additional conditions); Subgroup B, Subgroup 2 grouped with Subgroup 3; Subgroup C, all discopathies cases with or without concomitant disc herniation; Subgroup D, all osteochondrosis cases with or without concomitant disc herniation.

* P<0.05,

** P<0.01

^^^ P ≤ 0.10

**Table 3:** Association of lumbar spine pathologies and *VDR* BsmI, ApaI, and TaqI alleles.

	*B*	*b*	*A*	*a*	*T*	*t*
Controls n = 252 (%)	204/504 (40.5)	300/504 (59.5)	292/504 (57.9)	212/504 (42.1)	321/504 (63.7)	183/504 (36.3)
Cases n = 266 (%)	244/532 (45.9)^	288/532 (54.1)^	309/532 (58.1)	223/532 (41.9)	345/532 (64.8)	187/532 (35.1)
Subgroup 1 n = 88 (%)	84/176 (47.7)^	92/176 (52.3)^	103/176 (58.5)	73/176 (41.5)	114/176 (64.8)	62/176 (35.2)
Subgroup 2 n = 87 (%)	70/174 (40.2)	104/174 (59.8)	96/174 (55.2)	78/174 (44.8)	120/174 (69.0)	54/174 (31.0)
Subgroup 3 n = 40 (%)	41/80 (51.2)^	39/80 (48.8)^	52/80 (65.0)	28/80 (35.0)	45/80 (56.3)	35/80 (43.7)
Subgroup 4 n = 51 (%)	49/102 (48.0)	53/102 (52.0)	58/102 (56.9)	44/102 (43.1)	66/102 (64.7)	36/102 (35.3)
Subgroup 1+2+3 n = 215 (%)	195/430 (45.3)	235/430 (54.7)	251/430 (58.4)	179/430 (41.6)	279/430 (64.9)	151/430 (35.1)
Subgroup A n = 175 (%)	154/350 (44.0)	196/350 (56.0)	199/350 (56.9)	151/350 (43.1)	234/350 (66.9)	116/350 (33.1)
Subgroup B n = 127 (%)	111/254 (43.7)	143/254 (56.3)	148/254 (58.3)	106/254 (41.7)	165/254 (65.0)	89/254 (35.0)
Subgroup C n = 64 (%)	68/128 (53.1)*	60/128 (46.9)*	82/128 (64.1)	46/128 (35.9)	71/128 (55.5)^	57/128 (44.5)^
Subgroup D n = 50 (%)	32/100 (32.0)	68/100 (68.0)	48/100 (48.0)^	52/100 (52.0)^	79/100 (79.0)**	21/100 (21.0)**

Subgroup 1 = patients with disc herniation alone; Subgroup 2 = patients with discopathies and/or osteochondrosis associated with disc herniation; Subgroup 3 = patients with discopathies and/or osteochondrosis without herniation; Subgroup 4 = patients with stenosis and/or spondylolisthesis.

Subgroup A, Subgroup 1 grouped with Subgroup 2 (i.e. all hernia cases with or without concomitant additional conditions); Subgroup B, Subgroup 2 grouped with Subgroup 3; Subgroup C, all discopathies cases with or without concomitant disc herniation; Subgroup D, all osteochondrosis cases with or without concomitant disc herniation.

* P<0.05,

** P<0.01

^^^ P ≤ 0.10

By comparison of controls with all LDD cases, significant differences were observed for the heterozygous *Bb* and *Aa* genotypes (OR = 1.62, 95%CI = 1.11–2.36, P = 0.012, and OR = 1.60, 95%CI = 1.10–2.33, P = 0.014, respectively), while the homozygous *bb* genotype was protective (OR = 0.56, 95%CI = 0.37–0.84, P = 0.005).

By specific subgroups analysis, the *Bb* genotype was a risk factor for disc herniation (subgroup 1, OR = 1.97, 95%CI = 1.16–3.36, P = 0.013; subgroup 1+2+3, OR = 1.73, 95%CI = 1.16–2.56, P = 0.007; and subgroup A, OR = 1.67, 95%CI = 1.10–2.55, P = 0.017). On the contrary, the *bb* genotype was protective for almost all the subgroups (subgroup 1, OR = 0.40, 95%CI = 0.21–0.74, P = 0.004; subgroup 3, OR = 0.40, 95%CI = 0.17–0.95, P = 0.037; subgroup 1+2+3, OR = 0.54, 95%CI = 0.35–0.83, P = 0.005; subgroup A, OR = 0.57, 95%CI = 0.36–0.91, P = 0.018; and subgroup C, OR = 0.40, 95%CI = 0.20–0.82, P = 0.012). Patients with osteochondrosis with or without disc herniation showed an opposite trend, in fact, the *BB* genotype had a tendency to be protective (subgroup D, OR = 0.30, 95%CI = 0.09–1.08, P = 0.065).

The *Aa* genotype was a risk factor for disc herniation (subgroup 1, OR = 1.95, 95%CI = 1.15–3.32, P = 0.014; subgroup 1+2+3, OR = 1.76, 95%CI = 1.19–2.61, P = 0.005; and subgroup A, OR = 1.88, 95%CI = 1.23–2.88, P = 0.003). On the contrary, the *aa* genotype was protective for 2 subgroups (subgroup 1+2+3, OR = 0.52, 95%CI = 0.30–0.91, P = 0.021; subgroup A, OR = 0.55, 95%CI = 0.30–0.98, P = 0.043). For subgroup D (all osteochondrosis cases) the *Aa* genotype was a risk factor (OR = 2.70, 95%CI = 1.37–5.34, P = 0.004), while the *AA* genotype was protective (OR = 0.31, 95%CI = 0.13–0.73, P = 0.008). Finally, for the subgroup D only, the *tt* genotype was protective.

From the analysis of alleles distributions in pathological subgroups it was observed that *B* allele was a risk factor to develop discopathies with or without disc herniation (subgroup C, crude OR = 1.66, 95%CI = 1.10–2.51, P = 0.017), and the *T* allele was risky for all osteochondrosis (subgroup D, OR = 2.09, 95%CI = 1.21–3.61, P = 0.008).

For patients with stenosis and/or spondylolistesis no significant findings were obtained regarding genotypes and alleles.

### *VDR* BsmI, ApaI, TaqI haplotypes

**Fig 2:**
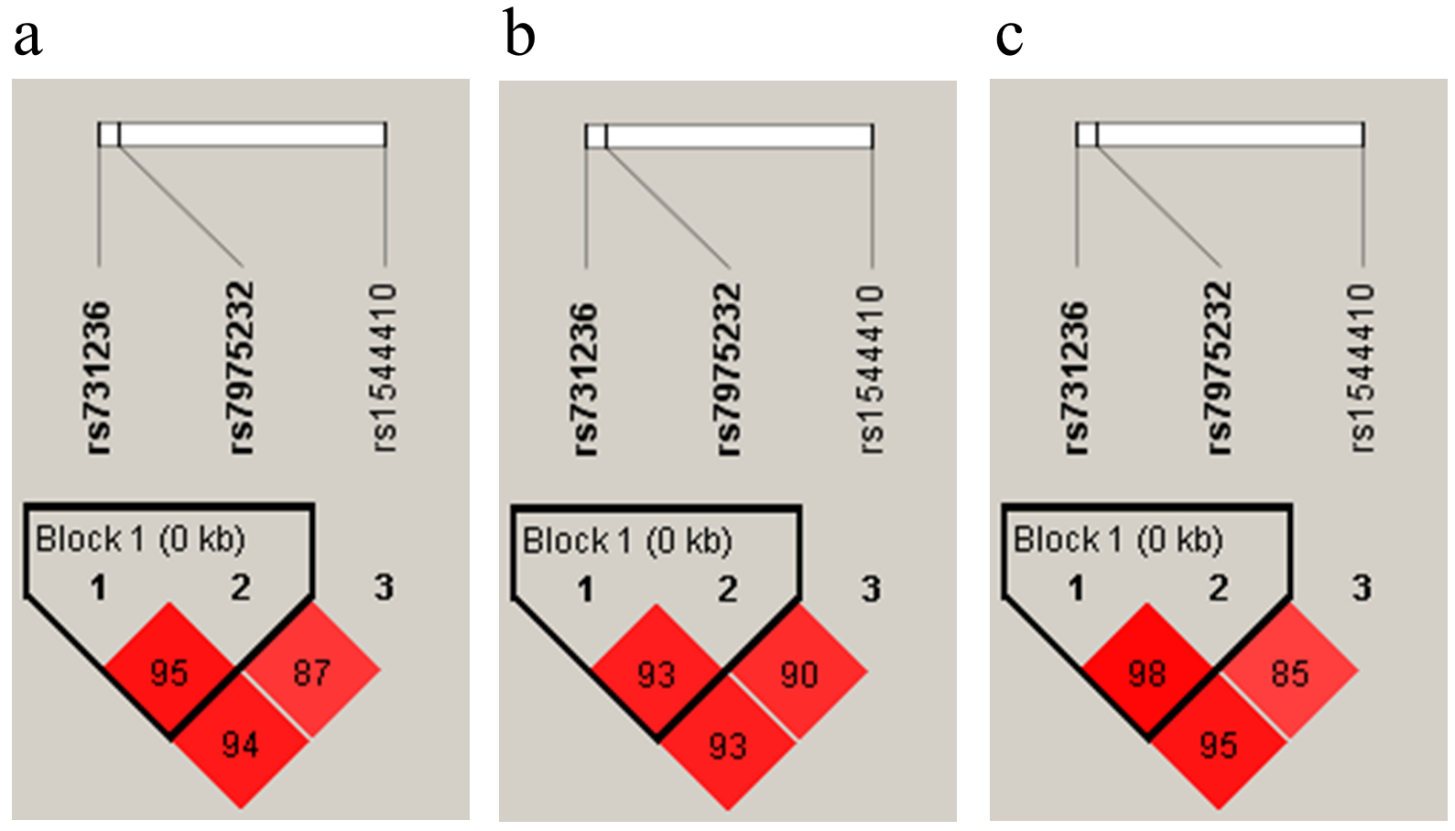
LD plots (r^2^) are shown for all subjects (a), controls (b) and cases (c).

LD plots (r^2^) showed that all the three SNPs were in linkage disequilibrium (Fig 2). The strongest LD was observed between ApaI and TaqI (r^2^ = 0.95 in all the subjects, r^2^ = 0.93 in controls, and r^2^ = 0.98 in cases). TaqI and BsmI showed slightly lower values of LD (r^2^ = 0.94 in all the subjects, r^2^ = 0.93 in controls, and r^2^ = 0.95 in cases), while BsmI and ApaI showed the lowest values (r^2^ = 0.87 in all the subjects, r^2^ = 0.90 in controls, r^2^ = 0.85 in cases).

Table 4 reported the frequencies of BsmI, ApaI and TaqI combined genotypes in controls compared with overall cases and specific subgroups of patients.

**Table 4:** Association of lumbar spine pathologies and *VDR* BsmI, ApaI, and TaqI combined genotypes.

Genotypes	*BbAaTt*	*bbaaTT*	*BBAAtt*	*BbAATt*	*bbAaTT*	*bbAATT*	*BBAATt*	*BbAaTT*	*BbAATT*	*BbaaTT*
Controls n = 252 (%)	66 (26.2)	47 (18.6)	34 (13.5)	29 (11.5)	28 (11.1)	13 (5.2)	10 (4.0)	9 (3.6)	5 (2.0)	4 (1.6)
Cases n = 266 (%)	79 (29.7)	29 (10.9)*	32 (12.0)	23 (8.6)	35 (13.2)	3 (1.1)*	13 (4.9)	26 (9.8)**	10 (3.8)	11 (4.1)^
Subgroup 1 n = 88 (%)	29 (32.9)	7 (7.9)*	10 (11.4)	6 (6.8)	11 (12.5)	1 (1.1)	5 (5.7)	11 (12.5)**	3 (3.4)	4 (4.5)
Subgroup 2 n = 87 (%)	26 (30.0)	12 (13.8)	8 (9.2)	4 (4.6)^	15 (17.2)	2 (2.3)	3 (3.4)	8 (9.2)*	4 (4.6)	2 (2.3)
Subgroup 3 n = 40 (%)	12 (30.0)	2 (5.0)*	7 (17.5)	6 (15.0)	5 (12.5)	0 (0)	2 (5.0)	3 (7.5)	1 (2.5)	1 (2.5)
Subgroup 4 n = 51 (%)	12 (23.5)	8 (15.7)	7 (13.7)	7 (13.7)	4 (7.8)	0 (0)	3 (5.9)	4 (7.8)^	2 (3.9)	4 (7.8)^
Subgroup 1+2+3 n = 215 (%)	67 (31.2)	21 (9.8)**	25 (11.6)	16 (7.4)	31 (14.4)	3 (1.4)^	10 (4.6)	22 (10.2)**	8 (3.7)	7 (3.2)
Subgroup A n = 175 (%)	55 (31.4)	19 (10.9)*	18 (10.3)	10 (5.7)^	26 (14.9)	3 (1.7)^	8 (4.6)	19 (10.9)**	7 (4.0)	6 (3.4)
Subgroup B n = 127 (%)	38 (29.9)	14 (11.0)^	15 (11.8)	10 (7.9)	20 (15.7)	2 (1.6)^	5 (3.9)	11 (8.7)*	5 (3.9)	3 (2.4)
Subgroup C n = 64 (%)	16 (25.0)	6 (9.4)^	14 (21.9)^	7 (10.9)	6 (9.4)	0 (0)^	2 (3.1)	5 (7.8)^	3 (4.7)	2 (3.1)
Subgroup D n = 50 (%)	17 (34.0)	8 (16.0)	0 (0)	1 (2.0)^	12 (24.0)*	1 (2.0)	3 (6.0)	5 (10.0)^	2 (4.0)	1 (2.0)

Subgroup 1 = patients with disc herniation alone; Subgroup 2 = patients with discopathies and/or osteochondrosis associated with disc herniation; Subgroup 3 = patients with discopathies and/or osteochondrosis without herniation; Subgroup 4 = patients with stenosis and/or spondylolisthesis.

Subgroup A, Subgroup 1 grouped with Subgroup 2 (i.e. all hernia cases with or without concomitant additional conditions); Subgroup B, Subgroup 2 grouped with Subgroup 3; Subgroup C, all discopathies cases with or without concomitant disc herniation; Subgroup D, all osteochondrosis cases with or without concomitant disc herniation.

* P<0.05,

** P<0.01

^^^ P ≤ 0.10

From the frequency distributions of the 10 identified combined genotypes we observed that the *BbAaTT* was 3-fold more frequent in all LDD cases (OR = 3.38, 95%CI = 1.49–7.67, P = 0.004), and had significant OR = 4.44, 95%CI = 1.62–12.2, P = 0.004 for subgroup 1; OR = 3.16, 95%CI = 1.07–9.33, P = 0.038 for subgroup 2; OR = 3.44, 95%CI = 1.48–7.97, P = 0.004 for subgroup 1+2+3; OR = 3.85, 95%CI = 1.60–9.28, P = 0.003 for subgroup A; and OR = 2.81, 95%CI = 1.07–7.42, P = 0.036 for subgroup B. The *bbaaTT* was protective for all cases (OR = 0.47, 95%CI = 0.27–0.82, P = 0.008), and specifically for subgroup 1, OR = 0.24, 95%CI = 0.09–0.66, P = 0.006; subgroup 3, OR = 0.20, 95%CI = 0.04–0.90, P = 0.036; subgroup 1+2+3, OR = 0.39, 95%CI = 0.21–0.72, P = 0.003; and subgroup A, OR = 0.43, 95%CI = 0.23–0.83, P = 0.012. The *bbAATT* was protective for all cases (OR = 0.22, 95%CI = 0.06–0.80, P = 0.022), and showed a protective tendency for all the discopathies, i.e. subgroup 1+2+3, OR = 0.27, P = 0.051, and for subgroup A, OR = 0.32, P = 0.086.

Finally, the *bbAaTT* was risky (OR = 2.56, 95%CI = 1.10–5.97, P = 0.030) only for subgroup D, i. e., for all patients with osteochondrosis, a subgroup with a peculiar pathological phenotype.

For patients with stenosis and/or spondylolistesis no significant findings were obtained regarding haplotypes.

Estimated haplotype frequencies were shown in Table 5. The most represented haplotype in the whole cohort of controls and cases was *baT*, followed by *BAt* and *bAT*.

**Table 5:** Estimated frequencies of *VDR* BsmI, ApaI, and TaqI haplotypes.

Haplotypes	*baT*	*BAt*	*bAT*	*BAT*	*bAt*	*BaT*
Controls n = 252	101 (40.4)	86 (34.4)	44 (17.6)	12 (4.8)	2 (1.1)	2 (1.0)
Cases n = 266	103 (38.9)	91 (34.3)	38 (14.4)	23 (8.8)	1 (0.6)	7 (2.8)
Subgroup 1 n = 88	34 (38.7)	30 (34.6)	11 (13.0)	9 (10.3)	0 (0.6)	2 (2.8)
Subgroup 2 n = 87	37 (42.7)	25 (29.8)	13 (15.9)	7 (8.3)	1 (1.2)	1 (2.2)
Subgroup 3 n = 40	12 (32.0)	16 (42.4)	6 (15.4)	2 (7.2)	0 (0)	0 (1.6)
Subgroup 4 n = 51	19 (35.6)	17 (35.3)	6 (13.4)	4 (8.2)	0 (0)	2 (4.6)
Subgroup 1+2+3 n = 215	83 (39.0)	73 (34.1)	31 (14.6)	19 (8.9)	1 (0.8)	5 (2.3)
Subgroup A n = 175	71 (40.6)	56 (32.2)	25 (14.4)	16 (9.3)	1 (0.9)	4 (2.5)
Subgroup B n = 127	49 (39.3)	42 (33.7)	19 (15.7)	10 (8.0)	1 (0.9)	2 (2.0)
Subgroup C n = 64	20 (32.2)	27 (42.8)	8 (13.0)	4 (7.4)	0 (0.9)	1 (2.9)
Subgroup D n = 50	25 (50.8)	10 (21.0)	8 (17.2)	4 (9.8)	0 (0)	0 (1.2)

Subgroup 1 = patients with disc herniation alone; Subgroup 2 = patients with discopathies and/or osteochondrosis associated with disc herniation; Subgroup 3 = patients with discopathies and/or osteochondrosis without herniation; Subgroup 4 = patients with stenosis and/or spondylolisthesis.

Subgroup A, Subgroup 1 grouped with Subgroup 2 (i.e. all hernia cases with or without concomitant additional conditions); Subgroup B, Subgroup 2 grouped with Subgroup 3; Subgroup C, all discopathies cases with or without concomitant disc herniation; Subgroup D, all osteochondrosis cases with or without concomitant disc herniation.

## Discussion

To the best of our knowledge, this is the first study demonstrating an association between the three *VDR* BsmI, ApaI and TaqI variants and clearly defined lumbar spine pathologies, and the only one study performed in the Italian population.

Genotype and allele frequencies were closed to those of Italian subjects from Central Italy reported in HapMap (101 subjects: genotypes *BB* = 17.8%, *Bb* = 48.5%, *bb* = 33.7%, alleles *B* = 42.1%, *b* = 57.9%; 102 subjects: genotypes *AA* = 36.3%, *Aa* = 45.1%, *aa* = 18.6%, alleles *A* = 58.8%, *a* = 41.2%; 101 subjects: genotypes *TT* = 33.7%, *Tt* = 48.5%, *tt* = 17.8%, alleles *T* = 57.9%, *t* = 42.1%) [29].

The distribution of genotypes and alleles in our cohort of controls was in accordance also with data of controls reported in a meta-analysis, based on studies published till December 2005, that investigated the association between these *VDR* polymorphisms and the risk of osteoporosis in Caucasian, East Asian, Mexican, Latino and Turkish women [30].

The heterozygous *Bb* and *Aa* genotypes represented a risk factor, while the homozygous *bb* genotype was protective for the all subjects with lumbar spine pathologies recruited in our study.

Other studies showed an association between BsmI, ApaI and TaqI *VDR* genotypes or alleles and signs of disc degeneration such as bulges, osteophytosis, disc space narrowing or disc herniation. In particular *B* allele in British [22], genotypes *TT* in Finnish [7], *Tt* and *tt* in Turkish [11,12] and Australian [14], *Tt* in Japanese [15] and alleles *t* and *A* in Chinese [17,19] patients were reported as predisposing towards worse phenotypes.

The inconsistent findings between our study and the data reported in literature [31] are likely related to both the ethnic differences among the study populations and to the different inclusion criteria, as well as to the lack of a standardized approach to define pathological phenotypes [32,33].

In our study, after the analysis of the overall cohort of LDD patients, we classified our cases in subgroups of clinical relevance, according to specific pathological features revealed by MRI. This approach allowed us to better define whether the associations of variants observed in all the pathological subjects were true for all the subgroups or only for particular lumbar spine pathologies.

The results were confirmed after adjusting for previously identified conventional low back pain risk factors [18]. Data observed for the overall cohort of subjects were confirmed for particular subgroups of patients. *Bb* genotype and *B* allele were associated with a 1.66 to 1.97-fold increased risk to develop disc herniation and discopathies concomitant or not with disc herniation. On the contrary, *bb* genotype was protective. *Aa* genotype was also associated with a 1.76 to 1.95-fold increased risk to develop discopathies and/or osteochondrosis associated or not with disc herniation, while *aa* genotype was protective.

Interestingly, the subgroup of patients with osteochondrosis showed particular features. *Aa* genotype was a risk factor (2.70-fold increased risk), while *AA* genotype was protective for this condition. In this subgroup of patients an opposite trend for BsmI was observed with respect to other patients, with a protective tendency for *BB* genotype. In the same subgroup, the *T* allele represented a 2.09-fold increased risk to develop osteochondrosis, while *tt* genotype was highly protective.

The three SNPs analyzed in our study were in high LD, where the greatest degree of LD was found between ApaI and TaqI, followed by TaqI and BsmI, and then BsmI and ApaI. A different trend was reported in the meta-analysis of Thakkinstian et al. [34], where the greatest degree of LD was observed between the BsmI and TaqI polymorphisms, followed by BsmI and ApaI, and then ApaI and TaqI. These discrepancies are likely related to the absence of HWE for all the three sites in one study [35] and for BsmI in another one [36] described in the meta-analysis, while our observed genotype frequencies were consistent with HWE.

The frequency distributions of the combined genotypes in our controls were close to those of non-osteoporotic postmenopausal women from Belgium [35], Italy [37], Denmark [36] and the Netherlands [38], and our estimated haplotype frequencies showed that the most common haplotype was *baT*, followed by *BAt* and *bAT* as reported for Caucasian women [34].

By excluding too rare combined genotypes (estimated to be less than 3% in frequency), we identified ten combined genotypes, among which notably the *BbAATT* had not been described before. Of these combinations, four were particularly interesting since they were associated with specific clinical conditions. The *BbAaTT* was risky (OR ranging from 2.81 to 4.44), while the *bbaaTT* and *bbAATT* were protective for patients with disc herniation and discopathies. As observed for genotypes and alleles, even for combined genotype analysis, the subgroup of patients with osteochondrosis showed peculiar features; in fact, the *bbAaTT* combined genotype had a 2.56-fold risk to develop osteochondrosis, a degenerative process involving primarily the vertebral bodies structures limiting the disc [39].

No significant findings were obtained regarding alleles, genotypes and haplotypes for patients with stenosis and/or spondylolistesis.

Interesting results were found for all the three SNPs BsmI, ApaI and TaqI, when analyzed in the light of clinical relevant phenotypes. In summary, patients affected by disc herniation and discopathies and/or osteochondrosis associated or not with disc herniation showed risky *B* allele, *Bb*, *Aa*, *BbAaTT* genotypes, and protective *bb*, *aa*, *bbaaTT*, *bbAATT* genotypes. In patients with osteochondrosis, *T* allele, *Aa*, *TT*, *bbAaTT* genotypes were risky, while *t* allele, *AA*, *tt* genotypes were protective.

The *bbaaTT* genotype, the main protective combined genotype for spine pathologies observed in our study, was more prevalent also in 144 non-osteoporotic Italian postmenopausal women compared to 176 osteoporotic subjects [37].

It is to note that, in our cohort, the *TT* genotype was risky or protective depending on the specific haplotype to which it belonged. This could explain the discrepancies between studies which evaluated only the TaqI polymorphism without a further haplotype analysis.

In the future, it will be our interest to enlarge the group of controls and the pathological subgroups to confirm present results in a larger cohort of subjects. This is particularly important since in large studies no association between these 3 *VDR* polymorphisms and the risk of osteoporosis [30], vertebral fracture incidence in women [40] and osteoarthritis in European population [41] was observed.

Moreover, basic science studies should be performed to reach a deeper knowledge of the molecular mechanism regulated by vitamin D through its receptor in the spine tissues. This will help to understand the association between these 3 SNPs in non-coding *VDR* sequence and spine pathologies. Particularly, it will be useful to study the interaction of BsmI, ApaI and TaqI *VDR* variants and other SNPs in nearby functional genes supposed to be in LD, such as collagen type II alpha 1 (*COL2A1*) [42], whose gene product is abundantly present in the intervertebral disc [43], and whose expression is regulated by vitamin D active metabolites [44].

Nevertheless our results highlighted the importance to perform a clear clinical evaluation of the patients with lumbar spine pathologies to obtain specific phenotypes for the identification of genetic markers of pathology. Moreover, the presence of specific genotypes/alleles/haplotypes associated with defined phenotypes suggests the possibility of a personalized clinical approach aimed to prevent or at least to delay the development of these spine disorders.

## Supporting Information

S1 Appendix Frequencies of BsmI, ApaI and TaqI genotypes in cases (1) and controls (0). (PDF)

S1 Fig Amplicon and digested fragments for BsmI *BB* genotype. Lanes 2 and 3 reported in Fig 1b.
(TIF)

S2 Fig Amplicon and digested fragments for BsmI *Bb* and *bb* genotypes. Lanes 12 and 13 and lanes 8 and 9, respectively, reported in Fig 1b.
(TIF)

S3 Fig Amplicon and digested fragments for ApaI *AA* and *Aa* genotypes. Lanes 14 and 16, and lanes 11 and 13, respectively, reported in Fig 1c.
(TIF)

S4 Fig Amplicon and digested fragments for ApaI *aa* genotype. Lanes 13 and 14 reported in Fig 1c.
(TIF)

S5 Fig Amplicon and digested fragments for TaqI *TT* and *tt* genotypes. Lanes 11 and 12 and lanes 5 and 6, respectively, reported in Fig 1d.
(TIF)

S6 Fig Amplicon and digested fragments for TaqI *Tt* genotype. Lanes 2 and 3 reported in Fig 1d.
(TIF)
