# Supplementary material for: BsmI, ApaI and TaqI Polymorphisms in the Vitamin D Receptor Gene (VDR) and Association with Lumbar Spine Pathologies: An Italian Case-Control Study
Source: PLoS One. 2016 May 5;11(5):e0155004. doi: 10.1371/journal.pone.0155004 (PMC4858196; doi:10.1371/journal.pone.0155004)
Supplement: S1 Appendix — (PDF) [file pone.0155004.s001.pdf]

ID	Subjects	BsmI	TaqI	Apa1
1	1	1,2	1,1	1,1
2	1	2,2	1,1	2,2
3	1	1,1	1,2	1,1
4	1	1,1	1,2	1,1
5	1	1,1	1,2	1,1
6	1	1,1	2,2	1,1
7	1	2,2	1,1	2,2
8	1	1,1	1,2	1,1
9	1	1,1	1,2	1,1
10	1	2,2	1,1	1,2
11	1	1,2	1,2	1,1
12	1	1,2	1,1	1,1
13	1	1,2	1,2	1,2
14	1	1,2	1,2	1,2
15	1	2,2	1,1	1,2
16	1	1,2	1,2	1,2
17	1	2,2	1,1	1,2
18	1	1,2	1,2	1,2
19	1	2,2	1,1	1,2
20	1	1,2	1,2	1,2
21	1	1,2	1,2	1,2
22	1	1,1	2,2	1,1
23	1	1,2	1,2	1,2
24	1	2,2	1,1	2,2
25	1	1,2	1,2	1,2
26	1	1,2	1,2	1,2
27	1	1,2	1,2	1,2
28	1	1,2	1,2	1,1
29	1	1,2	1,1	1,2
30	1	2,2	1,1	1,2
31	1	2,2	1,1	1,2
32	1	2,2	1,1	2,2
33	1	1,2	1,2	1,2
34	1	2,2	1,1	2,2
35	1	1,2	1,2	1,2
36	1	2,2	1,1	1,2
37	1	2,2	1,1	1,2
38	1	1,1	2,2	1,1
39	1	2,2	1,1	2,2
40	1	1,2	1,2	1,2
41	1	2,2	1,1	2,2
42	1	1,2	1,1	1,1
43	1	2,2	1,1	1,2
44	1	2,2	1,1	1,2
45	1	1,2	1,2	1,2
46	1	1,2	1,2	1,2
47	1	1,2	1,1	1,2
48	1	1,1	1,2	1,1
49	1	1,2	1,2	1,2
50	1	2,2	1,1	1,2
51	1	1,2	1,1	1,1
52	1	1,2	1,1	1,1

53	1	1,2	2,2	1,1
54	1	1,2	1,2	1,2
55	1	1,2	1,2	1,2
56	1	1,2	1,1	2,2
57	1	1,2	1,1	1,1
58	1	1,2	1,2	1,2
59	1	1,2	1,1	1,2
60	1	1,2	1,1	1,2
61	1	1,2	1,2	1,2
62	1	1,2	1,2	1,2
63	1	1,2	1,1	1,2
64	1	1,1	1,2	1,1
65	1	1,2	1,1	1,2
66	1	1,2	1,1	2,2
67	1	1,2	1,2	1,1
68	1	1,1	2,2	1,1
69	1	1,1	2,2	1,1
70	1	1,2	1,2	1,2
71	1	2,2	1,1	2,2
72	1	1,2	1,1	2,2
73	1	1,2	1,1	1,2
74	1	1,2	1,1	1,2
75	1	1,2	1,1	1,2
76	1	1,2	1,2	1,2
77	1	1,2	1,2	1,2
78	1	1,2	1,2	1,2
79	1	1,1	2,2	1,1
80	1	2,2	1,1	2,2
81	1	1,2	1,1	1,2
82	1	1,2	1,2	1,2
83	1	2,2	1,1	1,2
84	1	1,2	1,1	1,2
85	1	1,2	1,1	1,2
86	1	1,1	1,2	1,1
87	1	1,2	1,1	1,2
88	1	1,2	1,2	1,2
89	1	1,2	1,2	1,1
90	1	1,2	1,1	2,2
91	1	1,2	1,2	1,2
92	1	1,2	1,1	2,2
93	1	1,2	1,2	1,2
94	1	1,2	1,2	1,2
95	1	2,2	1,1	2,2
96	1	1,1	2,2	1,1
97	1	1,2	1,2	1,2
98	1	1,1	2,2	1,1
99	1	1,1	2,2	1,1
100	1	2,2	1,1	2,2
101	1	1,2	1,1	1,1
102	1	2,2	1,1	1,2
103	1	1,1	1,2	1,1
104	1	1,2	1,2	1,2
105	1	1,2	1,1	1,1

106	1	1,1	2,2	1,1
107	1	2,2	1,1	2,2
108	1	1,2	1,2	1,2
109	1	1,2	1,2	1,1
110	1	1,1	2,2	1,1
111	1	2,2	1,1	2,2
112	1	1,1	2,2	1,1
113	1	1,2	1,2	1,2
114	1	1,2	1,2	1,2
115	1	1,2	1,2	1,2
116	1	2,2	1,1	1,1
117	1	2,2	1,1	1,2
118	1	1,2	1,2	1,2
119	1	1,2	1,2	1,2
120	1	2,2	1,1	2,2
121	1	1,1	2,2	1,1
122	1	1,1	2,2	1,1
123	1	1,2	1,2	1,2
124	1	1,1	2,2	1,1
125	1	1,2	1,2	1,1
126	1	2,2	1,1	1,2
127	1	2,2	1,1	1,2
128	1	1,2	1,2	1,1
129	1	1,2	1,2	1,2
130	1	1,2	1,2	1,2
131	1	1,2	1,1	1,2
132	1	1,2	1,2	1,2
133	1	1,2	1,2	1,1
134	1	2,2	1,1	1,2
135	1	1,2	1,2	1,2
136	1	1,1	2,2	1,1
137	1	2,2	1,1	1,2
138	1	2,2	1,1	1,2
139	1	1,1	2,2	1,1
140	1	1,2	1,2	1,2
141	1	1,2	1,2	1,1
142	1	1,1	1,2	1,1
143	1	1,2	1,2	1,2
144	1	2,2	1,1	2,2
145	1	2,2	1,1	2,2
146	1	1,2	1,2	1,2
147	1	2,2	1,1	1,2
148	1	1,2	1,1	1,2
149	1	1,2	1,2	1,2
150	1	2,2	1,1	1,2
151	1	1,2	1,2	1,2
152	1	2,2	1,1	2,2
153	1	2,2	1,1	2,2
154	1	1,1	1,2	1,1
155	1	1,1	1,2	1,1
156	1	1,2	1,2	1,2
157	1	1,2	1,2	1,1
158	1	2,2	1,1	1,2

159	1	2,2	1,1	2,2
160	1	2,2	1,1	2,2
161	1	2,2	1,1	2,2
162	1	1,1	2,2	1,1
163	1	1,2	1,2	1,2
164	1	1,2	1,2	1,1
165	1	2,2	1,1	1,2
166	1	1,1	2,2	1,1
167	1	1,1	2,2	1,1
168	1	1,2	1,2	1,1
169	1	1,1	2,2	1,1
170	1	1,2	1,1	1,1
171	1	2,2	1,1	2,2
172	1	2,2	1,2	2,2
173	1	2,2	1,1	2,2
174	1	1,2	1,2	1,1
175	1	1,1	2,2	1,1
176	1	2,2	1,1	1,2
177	1	2,2	1,1	1,2
178	1	1,2	1,2	1,2
179	1	1,1	2,2	1,1
180	1	2,2	1,1	1,2
181	1	1,1	2,2	1,1
182	1	1,2	1,2	1,2
183	1	1,2	1,2	1,1
184	1	1,1	2,2	1,1
185	1	1,2	1,2	1,2
186	1	1,2	1,2	1,2
187	1	2,2	1,1	2,2
188	1	1,2	1,2	1,2
189	1	1,2	1,2	1,2
190	1	1,2	1,2	1,2
191	1	1,2	1,2	1,2
192	1	1,2	2,2	1,1
193	1	1,2	1,2	1,1
194	1	2,2	1,1	1,2
195	1	2,2	1,1	1,2
196	1	1,2	1,1	1,2
197	1	1,2	1,1	1,2
198	1	1,1	2,2	1,1
199	1	1,2	1,2	1,1
200	1	1,1	2,2	1,1
201	1	1,2	1,2	1,2
202	1	1,2	1,2	1,2
203	1	1,2	1,1	1,2
204	1	2,2	1,1	1,1
205	1	1,1	2,2	1,1
206	1	2,2	1,1	1,2
207	1	1,2	1,2	1,2
208	1	2,2	1,1	2,2
209	1	2,2	1,1	2,2
210	1	2,2	1,1	1,1
211	1	2,2	1,1	1,2

212	1	2,2	1,1	1,2
213	1	1,2	1,2	1,2
214	1	1,2	1,2	1,2
215	1	1,2	1,2	1,2
216	1	1,2	1,1	1,2
217	1	2,2	1,1	2,2
218	1	1,2	1,1	1,2
219	1	1,2	1,2	1,2
220	1	1,1	2,2	1,1
221	1	1,2	1,2	1,1
222	1	1,2	1,1	2,2
223	1	1,2	1,1	1,2
224	1	1,2	1,1	1,1
225	1	1,2	1,1	2,2
226	1	1,2	1,1	1,2
227	1	1,2	1,2	1,1
228	1	1,2	1,1	2,2
229	1	1,2	1,1	1,2
230	1	1,2	1,1	2,2
231	1	1,2	1,2	1,2
232	1	1,2	1,2	1,2
233	1	1,2	1,2	1,1
234	1	1,2	1,2	1,2
235	1	1,2	1,2	1,2
236	1	1,2	1,1	1,2
237	1	1,2	1,2	1,2
238	1	1,2	1,2	1,2
239	1	1,2	1,1	2,2
240	1	1,2	1,2	1,2
241	1	1,2	1,2	1,2
242	1	1,2	1,1	2,2
243	1	1,2	2,2	1,1
244	1	1,1	1,2	1,2
245	1	1,2	1,1	1,2
246	1	1,2	1,2	1,1
247	1	1,2	1,2	1,2
248	1	1,2	1,1	1,2
249	1	1,2	1,2	1,2
250	1	1,2	1,2	1,2
251	1	2,2	1,1	2,2
252	1	1,2	1,2	1,2
253	1	1,2	1,2	1,1
254	1	2,2	1,1	1,2
255	1	2,2	1,1	1,2
256	1	1,2	1,2	1,2
257	1	1,1	1,2	1,1
258	1	1,2	1,2	1,1
259	1	2,2	1,1	1,2
260	1	2,2	1,1	2,2
261	1	1,2	1,2	1,2
262	1	1,1	2,2	1,1
263	1	1,1	2,2	1,1
264	1	1,2	1,2	1,1

265	1	2,2	1,1	1,2
266	1	1,1	2,2	1,1
267	0	2,2	1,1	1,2
268	0	1,1	2,2	1,1
269	0	2,2	1,1	1,1
270	0	1,2	1,2	1,1
271	0	1,2	1,2	1,1
272	0	2,2	1,1	1,1
273	0	1,1	2,2	1,1
274	0	2,2	1,1	1,1
275	0	2,2	1,1	1,2
276	0	1,1	2,2	1,1
277	0	2,2	1,1	2,2
278	0	2,2	1,1	1,1
279	0	1,1	2,2	1,1
280	0	1,1	2,2	1,1
281	0	1,1	2,2	1,1
282	0	1,2	1,1	1,1
283	0	1,2	1,2	1,2
284	0	2,2	1,1	1,1
285	0	1,2	1,1	2,2
286	0	1,2	1,1	1,1
287	0	2,2	1,1	1,2
288	0	2,2	1,2	1,2
289	0	2,2	1,2	1,2
290	0	1,1	2,2	1,1
291	0	2,2	1,2	1,2
292	0	1,2	1,1	1,1
293	0	2,2	1,1	2,2
294	0	1,1	2,2	1,1
295	0	2,2	2,2	1,1
296	0	2,2	1,1	2,2
297	0	1,2	1,2	1,1
298	0	2,2	1,1	2,2
299	0	2,2	1,1	1,2
300	0	2,2	1,1	2,2
301	0	1,1	2,2	1,1
302	0	1,2	1,2	1,2
303	0	1,2	1,2	1,1
304	0	1,2	1,1	1,2
305	0	1,1	1,2	1,1
306	0	1,2	1,2	1,2
307	0	1,2	1,1	1,2
308	0	1,2	1,2	1,2
309	0	1,2	1,2	1,2
310	0	2,2	1,1	2,2
311	0	2,2	1,1	1,2
312	0	2,2	1,1	2,2
313	0	2,2	1,1	2,2
314	0	2,2	1,1	1,1
315	0	1,2	1,2	1,2
316	0	1,2	1,2	1,2
317	0	1,2	1,2	1,1

**318**	0	**1,2**	**1,2**	**1,1**
**319**	0	**1,1**	**1,2**	**1,1**
**320**	0	**1,1**	**2,2**	**1,1**
**321**	0	**2,2**	**1,1**	**2,2**
**322**	0	**1,2**	**1,1**	**1,2**
**323**	0	**2,2**	**1,1**	**2,2**
**324**	0	**1,1**	**1,2**	**1,1**
**325**	0	**2,2**	**1,1**	**1,2**
**326**	0	**2,2**	**1,1**	**2,2**
**327**	0	**1,1**	**1,2**	**1,1**
**328**	0	**1,2**	**1,2**	**1,1**
**329**	0	**1,1**	**2,2**	**1,1**
**330**	0	**1,2**	**1,2**	**1,1**
**331**	0	**2,2**	**1,1**	**2,2**
**332**	0	**2,2**	**1,1**	**2,2**
**333**	0	**1,2**	**1,2**	**1,2**
**334**	0	**2,2**	**1,1**	**1,1**
**335**	0	**2,2**	**1,1**	**1,2**
**336**	0	**1,2**	**1,1**	**2,2**
**337**	0	**2,2**	**1,1**	**1,2**
**338**	0	**1,1**	**2,2**	**1,1**
**339**	0	**2,2**	**1,1**	**1,1**
**340**	0	**1,2**	**1,2**	**1,2**
**341**	0	**1,2**	**1,2**	**1,1**
**342**	0	**1,2**	**1,2**	**1,2**
**343**	0	**2,2**	**1,1**	**1,2**
**344**	0	**1,2**	**1,1**	**1,2**
**345**	0	**2,2**	**1,1**	**1,2**
**346**	0	**2,2**	**1,2**	**1,2**
**347**	0	**1,2**	**1,2**	**1,1**
**348**	0	**2,2**	**1,1**	**2,2**
**349**	0	**1,1**	**2,2**	**1,1**
**350**	0	**2,2**	**1,1**	**1,2**
**351**	0	**1,1**	**2,2**	**1,1**
**352**	0	**1,2**	**1,2**	**1,2**
**353**	0	**1,2**	**1,1**	**2,2**
**354**	0	**1,2**	**1,2**	**1,1**
**355**	0	**2,2**	**1,1**	**1,2**
**356**	0	**2,2**	**1,1**	**2,2**
**357**	0	**1,1**	**2,2**	**2,2**
**358**	0	**2,2**	**1,1**	**1,1**
**359**	0	**1,2**	**1,2**	**1,2**
**360**	0	**1,2**	**1,2**	**1,2**
**361**	0	**2,2**	**1,1**	**2,2**
**362**	0	**1,2**	**1,2**	**1,2**
**363**	0	**2,2**	**1,1**	**2,2**
**364**	0	**1,2**	**1,2**	**1,2**
**365**	0	**1,2**	**1,2**	**1,1**
**366**	0	**1,1**	**2,2**	**1,1**
**367**	0	**1,2**	**1,2**	**1,2**
**368**	0	**1,2**	**1,2**	**1,1**
**369**	0	**1,1**	**2,2**	**1,1**
**370**	0	**1,2**	**1,2**	**1,2**

371	0	1,2	1,2	1,1
372	0	2,2	1,1	1,2
373	0	2,2	1,1	1,2
374	0	2,2	1,1	2,2
375	0	1,1	2,2	1,1
376	0	2,2	1,1	2,2
377	0	1,2	1,2	1,1
378	0	2,2	1,1	2,2
379	0	1,2	1,2	1,2
380	0	1,2	1,2	1,2
381	0	1,2	1,2	1,1
382	0	1,2	1,2	1,2
383	0	1,2	1,1	2,2
384	0	1,2	1,2	1,1
385	0	1,2	1,2	1,2
386	0	1,1	2,2	1,1
387	0	1,1	2,2	1,1
388	0	2,2	1,1	1,2
389	0	1,1	2,2	1,1
390	0	1,2	1,2	1,2
391	0	2,2	1,1	1,2
392	0	1,2	1,2	1,2
393	0	1,2	1,2	1,1
394	0	1,2	1,2	1,2
395	0	1,1	2,2	1,1
396	0	1,2	1,2	1,2
397	0	1,2	2,2	1,2
398	0	1,2	1,2	1,1
399	0	2,2	1,1	2,2
400	0	1,1	2,2	1,1
401	0	1,2	1,2	1,2
402	0	2,2	1,1	2,2
403	0	1,1	2,2	1,1
404	0	1,2	1,2	1,2
405	0	2,2	1,1	2,2
406	0	1,2	1,2	1,2
407	0	2,2	1,1	1,2
408	0	1,2	1,2	1,2
409	0	2,2	1,1	2,2
410	0	1,2	1,2	1,2
411	0	1,2	1,2	1,1
412	0	2,2	1,1	1,2
413	0	2,2	1,1	1,1
414	0	1,2	1,2	1,2
415	0	2,2	1,1	2,2
416	0	1,2	1,1	1,1
417	0	2,2	1,1	1,2
418	0	2,2	1,1	2,2
419	0	1,2	1,2	1,2
420	0	2,2	1,1	1,2
421	0	2,2	1,1	2,2
422	0	2,2	1,1	1,1
423	0	2,2	1,1	2,2

424	0	2,2	1,1	2,2
425	0	1,1	2,2	1,1
426	0	1,2	1,2	1,2
427	0	2,2	1,1	2,2
428	0	1,2	1,2	1,2
429	0	1,1	2,2	1,1
430	0	1,2	1,2	1,2
431	0	2,2	1,1	1,2
432	0	1,1	2,2	1,1
433	0	2,2	1,1	2,2
434	0	2,2	1,1	1,2
435	0	1,1	1,2	1,1
436	0	1,1	2,2	1,1
437	0	1,2	1,2	1,1
438	0	1,2	1,2	1,2
439	0	1,2	1,2	1,2
440	0	1,2	1,2	1,2
441	0	2,2	1,1	2,2
442	0	1,2	1,2	1,2
443	0	1,2	1,1	1,1
444	0	1,2	1,2	1,2
445	0	1,2	1,2	1,2
446	0	2,2	1,1	1,1
447	0	2,2	1,1	1,2
448	0	1,2	1,2	1,2
449	0	1,2	1,2	1,2
450	0	2,2	1,1	2,2
451	0	1,1	2,2	1,1
452	0	1,2	1,2	1,2
453	0	1,2	1,2	1,2
454	0	2,2	1,1	1,2
455	0	1,2	1,2	1,1
456	0	2,2	1,1	1,2
457	0	1,2	1,2	1,2
458	0	1,1	2,2	1,1
459	0	1,2	1,2	1,2
460	0	1,2	1,2	1,1
461	0	1,2	1,2	1,1
462	0	1,2	1,2	1,2
463	0	1,2	1,2	1,1
464	0	1,2	1,2	1,2
465	0	1,2	1,2	1,2
466	0	1,1	2,2	1,1
467	0	1,2	1,2	1,2
468	0	1,1	2,2	1,1
469	0	1,2	1,2	1,1
470	0	2,2	1,1	2,2
471	0	1,2	1,2	1,1
472	0	1,2	1,2	1,1
473	0	2,2	1,1	2,2
474	0	1,2	1,1	1,2
475	0	2,2	1,1	1,1
476	0	1,1	1,2	1,1

477	0	1,2	1,1	1,2
478	0	2,2	1,1	1,2
479	0	1,2	1,2	1,2
480	0	1,1	1,2	1,1
481	0	2,2	1,1	1,2
482	0	1,2	1,2	1,1
483	0	1,2	1,2	1,2
484	0	1,2	1,2	1,2
485	0	1,1	1,2	1,1
486	0	1,2	1,2	1,2
487	0	1,1	2,2	1,1
488	0	1,2	1,2	1,2
489	0	2,2	1,1	2,2
490	0	2,2	1,1	2,2
491	0	2,2	1,1	2,2
492	0	1,2	1,1	1,2
493	0	2,2	1,1	2,2
494	0	1,1	1,2	1,1
495	0	2,2	1,1	2,2
496	0	2,2	1,1	2,2
497	0	1,2	1,2	1,2
498	0	1,2	1,2	1,2
499	0	1,2	1,2	1,2
500	0	2,2	1,1	2,2
501	0	1,2	1,2	1,2
502	0	1,2	1,2	1,2
503	0	1,2	1,2	1,2
504	0	1,2	1,2	1,2
505	0	2,2	1,1	1,2
506	0	1,2	1,2	1,2
507	0	1,1	2,2	1,1
508	0	2,2	1,1	2,2
509	0	2,2	1,1	2,2
510	0	1,2	1,1	1,2
511	0	2,2	1,1	2,2
512	0	1,2	1,2	1,2
513	0	1,2	1,2	1,2
514	0	1,1	2,2	1,1
515	0	1,1	1,2	1,1
516	0	2,2	1,1	2,2
517	0	2,2	1,1	2,2
518	0	1,2	1,1	1,2
