# Supplementary figures and images for: BsmI, ApaI and TaqI Polymorphisms in the Vitamin D Receptor Gene (VDR) and Association with Lumbar Spine Pathologies: An Italian Case-Control Study
Source: PLoS One. 2016 May 5;11(5):e0155004. doi: 10.1371/journal.pone.0155004 (PMC4858196; doi:10.1371/journal.pone.0155004)

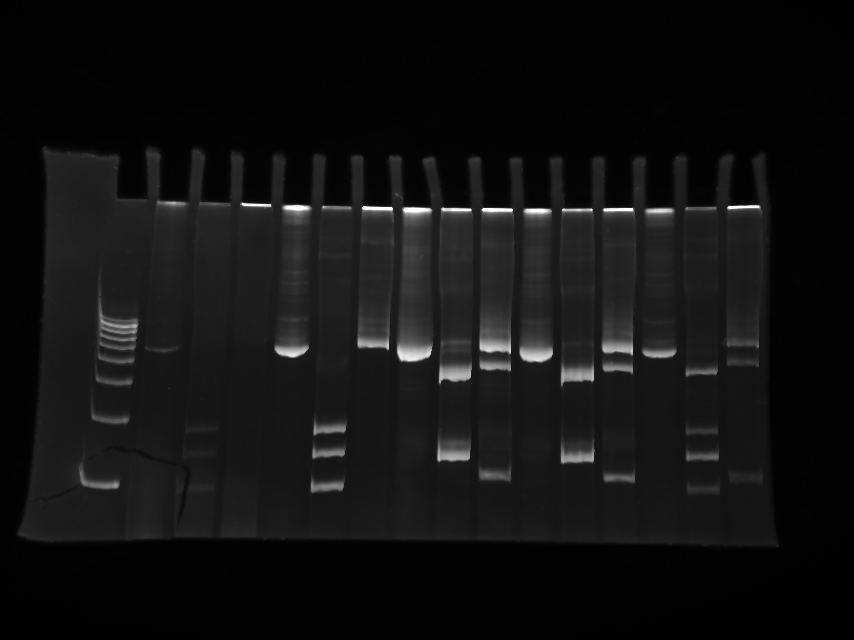

Supplement: S5 Fig — Lanes 11 and 12 and lanes 5 and 6, respectively, reported in Fig 1d. (TIF) [file pone.0155004.s006.tif]
